# Promotion of regulatory T cell induction by immunomodulatory herbal medicine licorice and its two constituents

**DOI:** 10.1038/srep14046

**Published:** 2015-09-15

**Authors:** Ao Guo, Dongming He, Hong-Bo Xu, Chang-An Geng, Jian Zhao

**Affiliations:** 1 School of Life Sciences, University of Science and Technology of China, Hefei, Anhui 230026, China; 2 Translational Medical Center for Stem Cell Therapy, Shanghai East Hospital, School of Medicine, Tongji University, Shanghai 200120, China; 3 State Key Laboratory of Phytochemistry and Plant Resources in West China, Kunming Institute of Botany, Chinese Academy of Sciences, Kunming 650201, China; 4 State Key Laboratory of Cell Biology, Institute of Biochemistry and Cell Biology, Shanghai Institute for Biological Sciences, Chinese Academy of Sciences, Shanghai 200031, China; 5 School of Life Science and Technology, ShanghaiTech University, Shanghai 201210, China

## Abstract

Regulatory T cells (Treg) play a critical role to control immune responses and to prevent autoimmunity, thus selective increase of Treg cells *in vivo* has broad therapeutic implications for autoimmune and inflammatory diseases. Licorice is a well-known herbal medicine used worldwide for over thousands of years, and accumulating evidence has shown its immunomodulatory potential. However, it is not clear whether licorice could regulate the induction and function of Treg cells. Here we found licorice extract could promote Treg cell induction, and then we used a rational approach to isolate its functional fractions and constituents. The results showed that two constituents, isoliquiritigenin and naringenin, promoted Treg cell induction both *in vitro* and *in vivo*. The effective fractions and two constituents of licorice also enhanced immune suppression of Treg cells, and they further reduced severity of DSS-induced colitis in mice. This study suggested that promotion of regulatory T cell induction could be an underlying mechanism of the historically and widely used herbal medicine licorice, providing its two effective molecules against autoimmune and inflammatory diseases.

Regulatory T (Treg) cells are a developmentally and functionally distinct CD4^+^ T cell subpopulation that is essential for maintaining immune tolerance and moderate inflammation induced by pathogens and environmental insults^1^,^2^. Treg cells comprise approximately 10% of peripheral CD4^+^ T cells and generate in the thymus^3^,^4^. However, peripheral naive CD4^+^ T cells can differentiate into induced Foxp3^+^ Treg cells in certain microenvironments, such as the gut^5^,^6^,^7^. Because of its pivotal role in modulating immune response and inflammation, there has been more interest in therapeutic manipulation of Treg cells to prevent autoimmune and limit chronic inflammatory diseases, such as inflammatory bowel disease (IBD)^8^,^9^. Forkhead box P3 (Foxp3) is a master regulator of Treg cell development and function. Foxp3 deficiency leads to systemic autoimmunity^1^,^10^,^11^,^12^. Other investigations showed activation of TGFβ-SMAD pathway is required for Treg cell generation from naive mouse CD4^+^ T cells^13^. However, a growing data has revealed that other pathways contribute to the regulation of Treg cell differentiation, including the downstream signaling of T cell receptor (TCR)^14^,^15^. Notably, inhibition of AKT and mTOR signaling, lead to Foxp3 expression upon TCR stimulation and promote Treg cells differentiation^15^,^16^,^17^,^18^.

Licorice, the root of Glycyrrhiza species, is one of the oldest and most popular herbal medicines used in many Asian and European countries for over 4000 years^19^. It is known as a well-recognized medicine against peptic ulcer disease, constipation, cough and viral infection^7^,^8^. Glycyrrhizin and flavonoids such as liquiritin, isoliquiritin, and their aglycones have been reported as the major constituents of licorice^20^. Licorice and its constituent, isoliquiritigenin, were reported to inhibit LPS-induced NF-kB activation and NLRP3 inflammasomes activation^21^,^22^,^23^. Glycyrrhizin inhibits tissue inflammation by reducing reactive oxygen species (ROS) generation by neutrophils^24^. In another study, licorice was believed to be involved in COX-2 inhibition and reducing prostaglandin (PGE2) which play a role in repression of inflammation^23^,^25^. Those investigations show that licorice has a significant anti-inflammatory properties *in vitro* and *in vivo* through multiple mechanisms. But whether licorice and its constituents could regulate the Treg cells generation and function is not clear.

Here, we explored the activity of licorice in Treg cell differentiation and function. By fractionation and tracing the Treg cell-inducing activity, we found isoliquiritigenin and naringenin, two constituents of licorice, increase Treg cell differentiation.

## Result

### Licorice extract promote regulatory T cells differentiation *in vitro*

**Figure 1:**
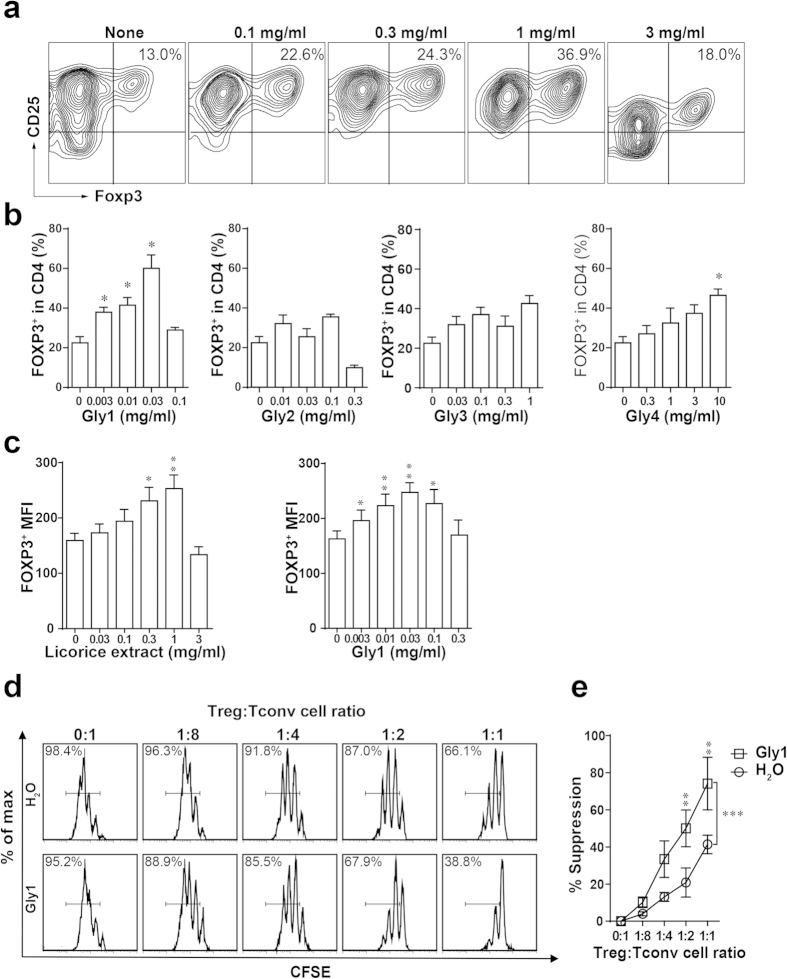
Licorice extract and its fractions promote Treg cell induction *in vitro.* (**a**) Naive CD4^+^ T cells were stimulated with immobilized anti-CD3, soluble anti-CD28 monoclonal antibodies and TGFβ (1 μg/ml) under the indicated concentrations of licorice extract and analyzed by FACS. (**b**) Naive CD4^+^ T cell were stimulated as in (**a**) under the indicated licorice fractions and analyzed by FACS. (**c**) Analysis of Foxp3 protein expression of a per-cell basis in Treg cells generated in the presence of licorice extract or Gly1 fraction treatment. Data are shown as mean fluorescence intensity (MFI). (**d**,**e**) CD4^+^CD25^+^ Treg cells untreated or treated with Gly1 were incubated with CFSE labelled CD4^+^CD25^−^ conventional T cells in an *in vitro* suppression assay. The suppression was assayed by FACS analysis for dilution of CFSE in gated conventional T cells. Results are expressed as means ± SEM and are representative of more than three experiments. *P < 0.05, **P < 0.01 and ***P < 0.001, as determined by one-way ANOVA followed by Bonferroni’s test.

The *in vitro* T cell differentiation assay was carried out to examine whether the tested traditional Chinese medicine extracts could increase the generation of Foxp3^+^ regulatory T cells. Indeed, we found that extract of licorice, an immunomodulatory traditional Chinese medicine, potentiated induction of Foxp3 after stimulation of purified naive (CD4^+^CD25^−^) T cells by CD3 and CD28 antibodies and transforming growth factor-beta (TGFβ)(Fig. 1a and Supplementary Fig. 1). The effect was dose dependent and an optimum of Treg cell induction was achieved by adding 1 mg/ml licorice extract in the presence of Treg-inducing cytokines. We also examined the effects of licorice extract on Th17 and Th1 cell differentiation *in vitro*, the percent of IL-17-and IFN-γ-expressing cells were not changed in Th17-or Th1-incuction conditions (Supplementary Fig. 2). These results indicated that licorice extract specific promote Foxp3^+^ Treg cell induction, but not Th1 or Th17 cell induction.

### Licorice fractions promote regulatory T cell differentiation and function *in vitro*

To identify the active ingredient in licorice, the extract of licorice was fractionated into four fractions and tested for the activity on the induction of Treg cells *in vitro*. Naive CD4^+^ T cells were treated with these licorice fractions in Treg-inducing condition, and CD4^+^CD25^+^Foxp3^+^ Treg cells were monitored. We found Gly1 fraction significantly increased the numbers of Treg cells in purified naive CD4^+^ T cells stimulated by CD3 and CD28 antibody and TGFβ. This effect was also dose dependent and an optimum of Treg cell induction was achieved by adding 0.03 mg/ml Gly1 (Fig. 1b). In our experiment, the Gly4 fraction also exhibited potential in promoting the induction of Foxp3^+^ Treg cells, although the effect was weaker than the Gly1 fraction (Fig. 1b). We analyzed the composition of Gly4 fraction, and found glycyrrhizic acid was abundant in it. As reported, glycyrrhizic acid has a potential to enhance Treg cells in lung of ovalbumin-sensitized mice^26^. Changes of Th1 and Th17 cells were not observed in their inducing conditions with licorice fractions (Supplementary Fig. 2). We also analyzed the Th1 and Th17 cytokines expression on CD4^+^ T cells with licorice and Gly1 fraction treatment. The result showed that most Th1 and Th17 cytokines, including IFNγ, TNF-α, IL17A, IL-17F, IL-21 and IL-22, expression was not changed (Supplementary Fig. 4). But we found IL-2, which was produced by Th1 cells and promoting T cells proliferation and survival, was reduced with licorice or Gly1 treatment. It indicated that licorice might suppress inflammation by reducing the expression of IL-2.

In addition to increasing the number of Treg cells, we found that Treg cells generated in the presence of licorice fraction Gly1 *in vitro* expressed higher amounts of Foxp3 protein on a per-cell basis than those from licorice extract-free cultures (Fig. 1c). It has been reported Foxp3 was a key regulatory factor in not only Treg cell differentiation, but also Treg cell function to suppress immune response^10^. The high level of Foxp3 expression indicated Treg cells induced by licorice extract and its active fraction might have an enhanced function. To verify whether the licorice active fraction Gly1 improved Treg cell function, Treg cells treated with or without Gly1 fraction were co-cultured with conventional T cells (Tconv) and antigen present cells. Proliferation of Tconv cells were analyzed after 4 days by FACS. Compared with Treg cells without treatment, Treg cells treated with Gly1 fraction displayed enhanced suppressive function toward Tconv cells proliferation (Fig. 1d,e). Thus, licorice extract and its active fraction Gly1 both promote Treg cells induction and function *in vitro*.

### Licorice and its fraction promote regulatory T cell induction *in vivo*

**Figure 2:**
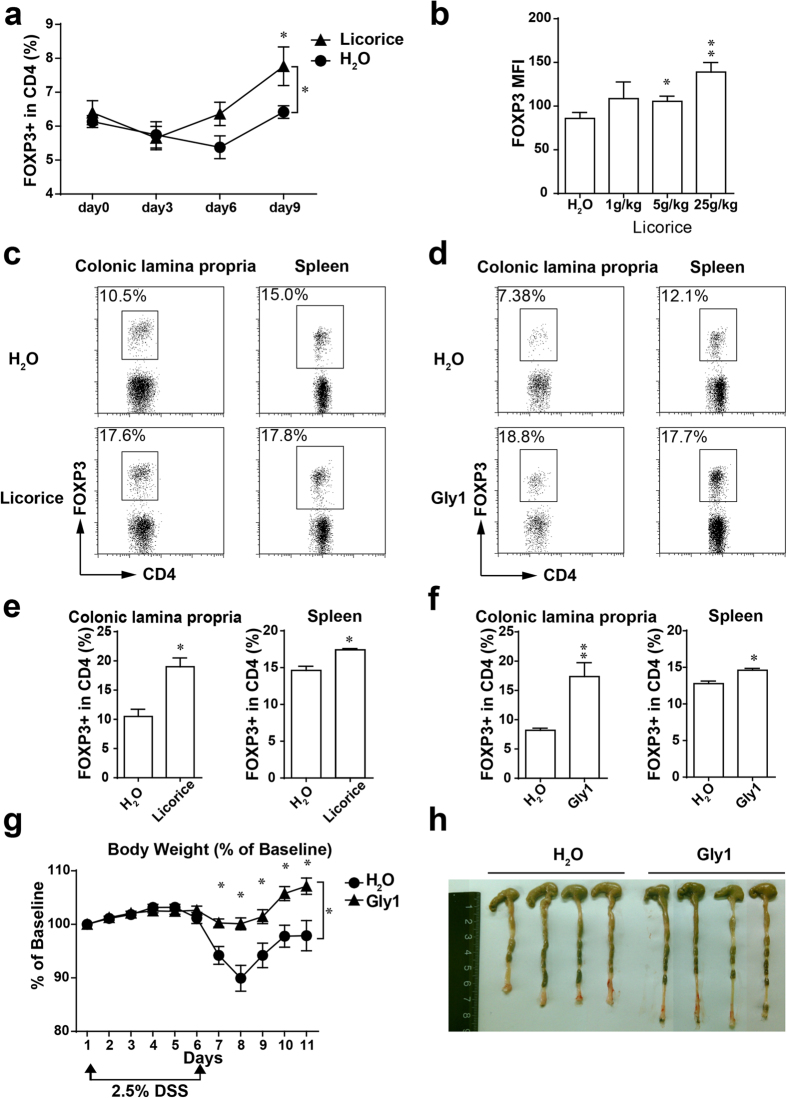
Licorice extract and its active fraction Gly1 promote Treg cells *in vivo*. (**a**) C57BL/6 mice were orally administrated with licorice or water, Foxp3^+^CD4^+^ Treg cells in peripheral blood were monitored every three days (n = 5 mice per group). (**b**) Analysis of Foxp3 protein expression of a per-cell basis in (**a**). Data are shown as mean fluorescence intensity (MFI). (**c**–**f**) C57BL/6 mice were orally administrated with licorice (**c**) or Gly1 fraction (**d**), Foxp3^+^CD4^+^ Treg cells in colonic lamina propria and spleen were analyzed after two weeks (n = 5 mice per group). (**e**) Quantification of the result in (**c**) (n = 5 mice per group). (**f**) Quantification of the result in (**d**). (**g**,**h**) Mice were treated with Gly1 fraction and induced colitis using dextran sulfate sodium, and the body weight (**g**) and colon shortness (**h**) were analyzed (n = 6 mice per group). Results are expressed as means ± SEM and are representative of three experiments. *P < 0.05, **P < 0.01 and ***P < 0.001, as determined by Man-Whitney U test (**e**,**f**), one-way ANOVA followed by Bonferroni’s test (**b**), or two-way ANOVA (**a**,**g**).

To corroborate the role of licorice extract in Treg cell induction *in vivo*, specific pathogen-free (SPF) C57BL/6 mice were orally administered with licorice extract and monitored the Foxp3^+^ population of Treg cells in the peripheral blood every three days, and we detected an increased Treg cell percentage in the peripheral blood (Fig. 2a,b). Then we monitored the Treg cells in spleen, lymph node and colonic lamina propria after two weeks treated with licorice extract. Although we detected only very modest changes in the splenic and lymph node Treg cell subsets, provision of licorice extract to mice resulted in a robust increase in colonic lamina propria Treg cells *in vivo* (Fig. 2c,e). Consistent with the result *in vitro*, Th1 and Th17 cells were not changed *in vivo*, which suggested licorice and its fraction specifically promote Treg cell induction (Supplementary Fig. 3). To determine whether the Gly1 fraction could increase Treg cells induction *in vivo* like total licorice extract, we orally administrated Gly1 fraction to C57BL/6 mice and monitored the Treg cells in spleen, lymph node and colonic lamina propria. Consistent with the total extract of licorice, colonic Treg cells were significantly augmented with Gly1 fraction administrated, whereas it was slightly in spleen and lymph node (Fig. 2d,f).

As we known, Regulatory T cells are critical in the prevention of inflammatory diseases. Selective increase of Treg cells *in vivo* could control inflammatory responses and have broad therapeutic implications^8^,^27^,^28^. As licorice extract and Gly1 fraction generated Treg cells more significant in colon, we sought to investigate the possibility that Gly1 would be efficacious for colitis, potentiating its application as a treatment for inflammatory colitis. Animals were induced for DSS induced inflammatory bowel disease (IBD), and groups were treated with water or Gly1 fraction by oral administration. Water-treated DSS induced animals lost a significant amount of weight by day 8, whereas Gly1 treatment significantly reduced the symptoms of DSS-induced IBD, such as weight loss and colon shortening were significantly suppressed in Gly1 treated groups (Fig. 2g,h).

### Isoliquiritigenin and naringenin are two active constituents of licorice to promote Treg cell induction and function

**Figure 3:**
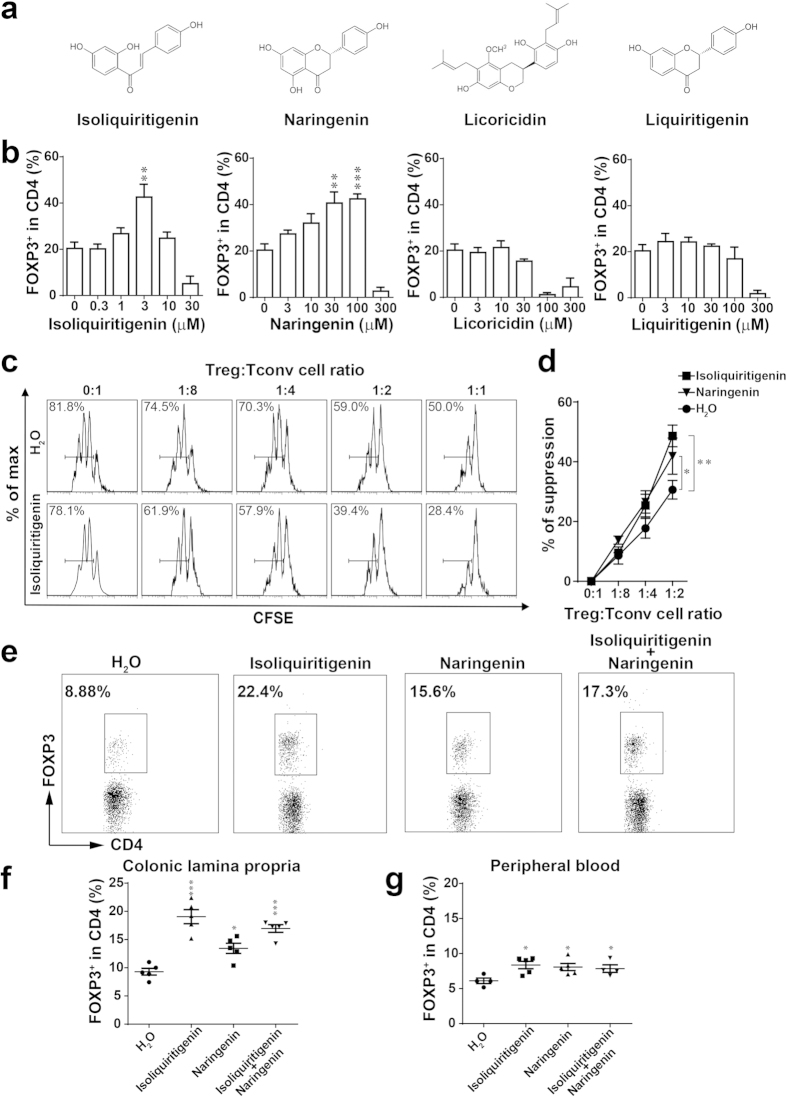
Isoliquiritigenin and naringenin are two active constituents of licorice to promote Treg induction and function. (**a**) Structures of four major constituents from Gly1 fraction. (**b**) Naive CD4^+^ T cells were stimulated with Treg-inducing conditions in the absence or presence of isoliquiritigenin, naringenin, licoricidin, liquiritigenin. Foxp3^+^CD4^+^ Treg cells were analyzed by FACS. (**c**) CD4^+^CD25^+^ Treg cells untreated or treated with isoliquiritigenin were incubated with CFSE labelled CD4^+^CD25^−^ conventional T cells. The suppression was assayed by FACS analysis for dilution of CFSE in gated Tconv cells. (**d**) Quantification of the result in (**c**). (**e**–**g**) C57BL/6 mice were orally administrated with isoliquiritigenin or naringenin for two weeks, colonic lamina propria Treg cells (**e**,**f**) and peripheral blood Treg cells (**g**) were monitored (n = 5 mice per group). Results are expressed as means ± SEM and are representative of more than three experiments. *P < 0.05, **P < 0.01 and ***P < 0.001, as determined by One-way ANOVA followed by Bonferroni’s test (**b**,**f**,**g**), or two-way ANOVA (**d**).

To identify the active constituents with Treg cell-inducing activity, we fractionated the Gly1 fraction into four sub-fractions and tracing the Treg cell-inducing activity of those sub-fractions on *in vitro* Treg cell differentiation assay. As a result, only the Gly18 and Gly19, two sub-fraction of Gly1, had the ability to promote Treg cell induction and function (Supplementary Fig. 5). Then the chemical composition of Gly18 and Gly19 sub-fraction was analyzed by thin layer chromatography (TLC) and NMR (Supplementary Fig. 6 and Supplementary Fig. 7). Four constituents, liquiritigenin, isoliquiritigenin, naringenin and licoricidin were found in these fractions (Fig. 3a)^29^,^30^. Recent study reported that naringenin could affect Treg cells induction *in vitro*, but the Treg cells inducing capability of other three chemicals were still unexplored^31^. We wanted to know whether other three chemicals could also increase Treg cell generation. So we examined Treg cell induction by these four chemicals *in vitro*. Indeed, isoliquiritigenin and naringenin were found to increase Treg cells differentiation in Treg cell inducing condition *in vitro*, and the other two chemicals didn’t exhibit the activity of promoting Treg cells (Fig. 3b).

Next, we examined whether isoliquiritigenin and naringenin could enhance Treg cell function. CD4^+^ naive T cells were isolated from spleen of wild type C57BL/6 mice and labeled with CFSE, then co-cultured with Treg cells treated with or without isoliquiritigenin and naringenin. Finally, we found that Tconv cells co-cultured with Treg cells treated with these two chemicals showed lower proliferation than which co-cultured with Treg cells without treatment (Fig. 3c,d). These results showed isoliquiritigenin and naringenin not only promote Treg cells differentiation, but also enhanced Treg cells function to suppress effector T cells proliferation.

### Isoliquiritigenin and naringenin promote Treg cells *in vivo* and attenuate DSS induced colitis

**Figure 4:**
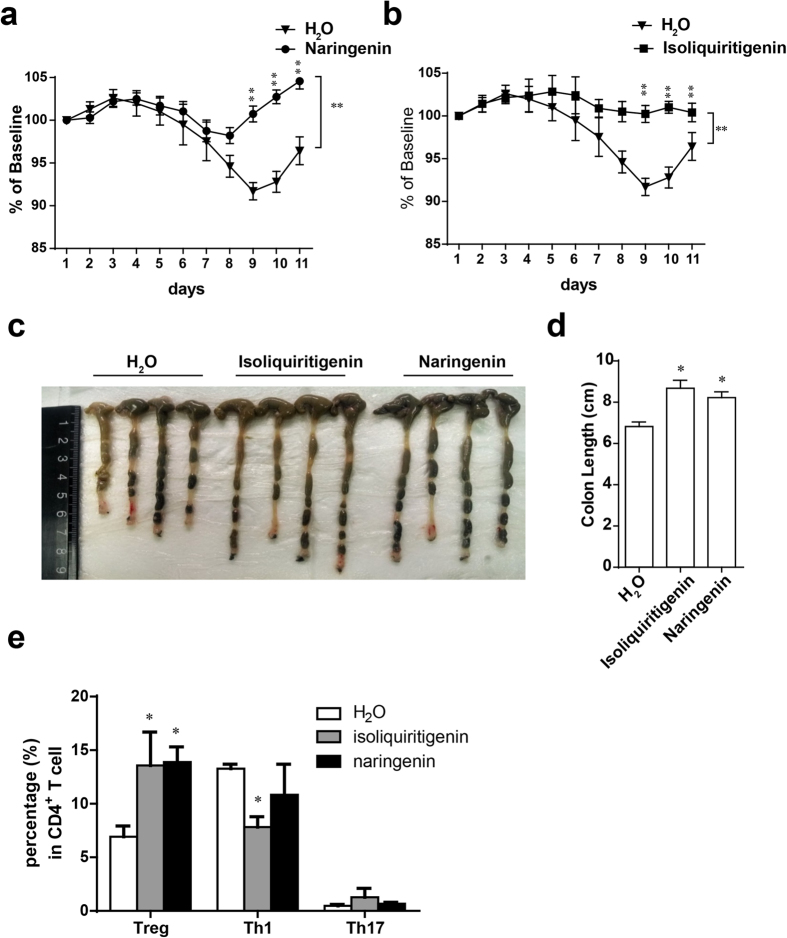
Isoliquiritigenin and naringenin attenuate DSS induced IBD. C57BL/6 mice were given 2.5% (w/v) DSS in drinking water for 6 days. Isoliquiritigenin and naringenin were orally administrated every day, starting 7 days prior to the DSS treatment (n = 5 mice for each group). (**a**,**b**) Body weight of isoliquiritigenin (**a**) or naringenin (**b**) treated mice with the DSS-induced colitis. (**c**,**d**) Colon length of mice treated with isoliquiritigenin or naringenin and control mice. (**e**) FACS profile of colonic lamina propria Treg, Th17 and Th1 cells isolated from mice treated with isoliquiritigenin, naringenin or water. Results are expressed as means ± SEM and are representative of more than three experiments. *P < 0.05, **P < 0.01 and ***P < 0.001, as determined by one-way ANOVA followed by Bonferroni’s test (**d**,**e**), or two-way ANOVA (**a**,**b**).

To confirm that isoliquiritigenin and naringenin promote Treg cells *in vivo*, wild type C57BL/6 mice were orally administrated with isoliquiritigenin, naringenin, or water. As expected, increased Treg cells were observed in peripheral blood and colonic lamina propria after two weeks of treatment, but the Th1 and Th17 cell change were not observed (Fig. 3e–g and Supplementary Fig. 9). Then we tested these two chemicals in DSS-induced mouse model of IBD. Animals were induced by 2.5% DSS in drinking water and isoliquiritigenin, naringenin or water were oral administrated every day. Compared with water-treated group, the symptoms of colitis, such as weight loss and rectal bleeding, colon shortening were significantly suppressed in isoliquiritigenin or naringenin treated groups (Fig. 4a–d). And an increase of Treg cells was observed in colonic lamina propria of isoliquiritigenin or naringenin treated groups (Fig. 4e). Interestingly a reduced number of Th1 cells was also observed in isoliquiritigenin treated group (Fig. 4e). These results suggested isoliquiritigenin and naringenin had therapeutic potential for inflammatory bowel disease.

### Isoliquiritigenin and naringenin promote Treg cells by affect TCR-Akt-mTOR and AhR signaling

**Figure 5:**
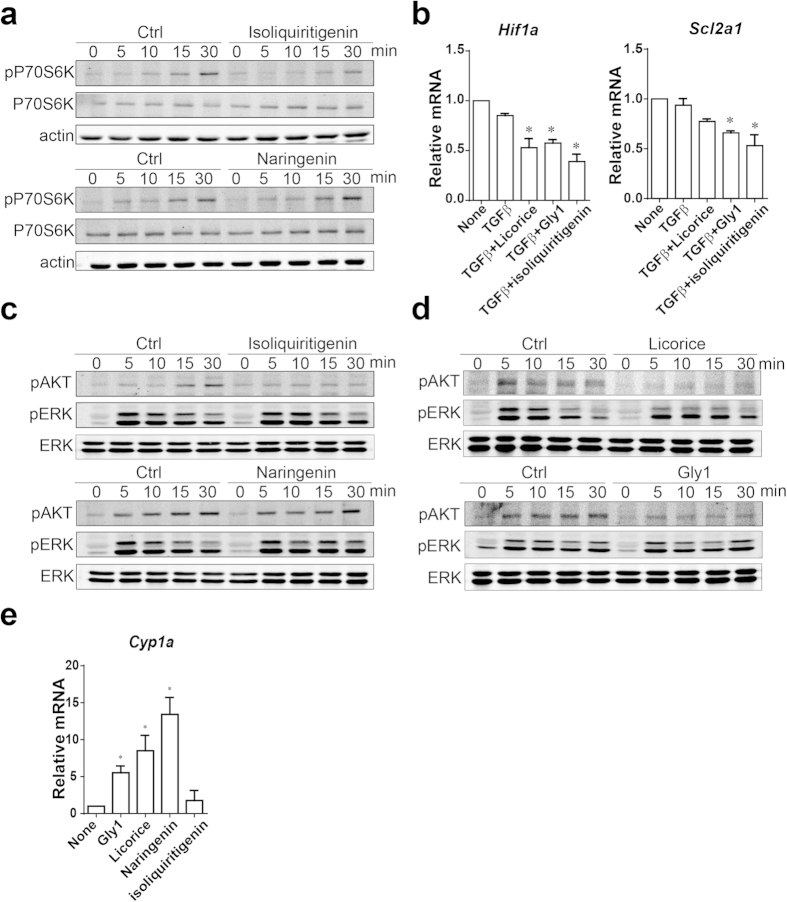
Isoliquiritigenin reduces Akt-mTOR signaling pathway activity. (**a**) Immunoblotting of phosphorylation P70 S6 kinase and total P70 S6 kinase in CD4^+^ T cells under Treg-inducing conditions and treated with isoliquiritigenin or naringenin for indicated times. (**b**) Quantitative PCR analyses of gene expressions in CD4^+^ T cells under Treg-inducing conditions treated with isoliquiritigenin or naringenin. (**c**) Immunoblot of phosphorylation Akt and Erk in CD4^+^ T cells under Treg-inducing conditions and treated with isoliquiritigenin or naringenin for indicated times. (**d**) Immunoblotting of phosphorylation Akt and Erk in CD4^+^ T cells under Treg-inducing conditions and treated with licorice or Gly1 fraction for indicated times. (**e**) Quantitative PCR analyses of *Cyp1a* expression in CD4^+^ T cells treated with licorice, Gly1 fraction, isoliquiritigenin or naringenin. The immunoblot in (**a**,**c**,**d**) were run under the same experimental conditions. Cropped blots were shown in (**a**,**c**,**d**) and the full-length blots were presented in Supplementary Figure 10. Results are expressed as means ± SEM and are representative of more than three experiments. *P < 0.05, **P < 0.01 and ***P < 0.001, as determined by one-way ANOVA followed by Bonferroni’s test (**b**,**e**).

The aforementioned findings suggested that isoliquiritigenin and naringenin were agents effective in regulating Treg cell induction and function to suppress the immune response. We sought to further understand the molecular mechanism underlying those effects on Treg cells. The aryl hydrocarbon receptor (AHR), a transcription factor mediating xenobiotic detoxification, plays an important role in controlling Treg cells generation^32^, and recent study reported that naringenin promote Treg cells as an AHR agonist^31^. So we wanted to know whether licorice, its active fraction and its constituent, isoliquiritigenin promote Treg cells also by AHR signaling. We cultured the naive CD4^+^ T cells in presence of licorice, Gly1 fraction, isoliquiritigenin or naringenin for 24 hr and AHR indicator gene *Cyp1a* was detected by real-time PCR. Naive CD4 T cells cultured with naringenin, licorice extract or Gly1 fraction were found an increased expression of CYP1A as expected (Fig. 5e). But different with naringenin, the increased expression of CYP1A was not obvious in naive CD4 T cells cultured with isoliquiritigenin (Fig. 5e). That result suggested isoliquiritigenin might control Treg cells generation in a different way from naringenin.

The mTOR pathway is important in regulating Th cells and Treg cells differentiation^11^,^16^,^33^. Inhibition of the mTOR pathway leads to enhanced Treg cells generation, an observation similar to isoliquiritigenin-mediated effect. We thus hypothesized that isoliquiritigenin affected the mTOR signaling in T cells to control Treg cells generation. Indeed, by assessing the phosphorylation of P70S6K, an indicator for the activation of the mTOR signaling, we found that isoliquiritigenin treatment reduced mTOR signaling activity in response to TCR activation (Fig. 5a). Notably, the mRNA expression of Treg-related mTOR downstream genes, such as hypoxia-inducible factor 1 (*Hif1*α) and glucose transporter 1 (*Slc2a1*) was also lower in CD4 T cells treated with isoliquiritigenin (Fig. 5b)^34^. This decrease was also observed in T cells treated with Licorice extract or Gly1 fraction (Fig. 5b).

The reduction in p70S6K phosphorylation in response to TCR by isoliquiritigenin suggested that isoliquiritigenin interfered TCR- and mTOR-mediated pathway. One of the major molecules in that pathways downstream of TCR is the AKT. Previous research has reported TCR controlling Foxp3 expression and Treg generation via Akt^15^,^16^, and isoliquiritigenin was reported as an inhibitor of Akt in cancer cells^35^. To evaluating whether Akt activation on response to TCR activation was affected by isoliquiritigenin, we measuring the phosphorylation of Akt upon CD3 and CD28 antibody stimulation in the presence or absence of isoliquiritigenin. The significantly reduced Akt phosphorylation was observed in isoliquiritigenin treated CD4 T cells, whereas the phosphorylation change of ERK was not observed (Fig. 5c). Consistent with isoliquiritigenin, licorice and its active fraction Gly1 also reduced the Akt activation upon TCR stimulation (Fig. 5d). These results suggest licorice, its active fraction Gly1 and active constituent isoliquiritigenin could attenuate TCR-Akt-mTOR axis and promote Treg cell generation.

## Discussion

Our data presented in this study demonstrate that licorice, an old and widely used herbal medicine, could increase the induction of Treg cells *in vitro* and *in vivo*. These findings suggested that promotion of regulatory T cell induction could be an underlying mechanism of licorice to modulate immune response. So far, more than 400 compounds have been isolated from licorice^20^,^36^. Triterpene saponins and flavonoids are believed to be responsible for the bioactivities of licorice^20^,^37^. It has been believed glycyrrhizic acid and its aglycone glycyrrhetic acid are the only pharmacological active constituent of licorice for a long time^37^. In recent years, licorice flavonoid are more and more popular because of their significant bio-activity in antimicrobial, antioxidative, and anti-inflammatory function. Isoliquiritigenin and naringenin are two important flavonoids in licorice. The concentration of isoliquiritigenin is about 9 mg/g in licorice extract, and naringenin in licorice is much less than isoliquiritigenin^38^. In our study, we found isoliquiritigenin and naringenin showed a notable capacity for promoting Treg cell induction. Compared with glycyrrhizic acid which was reported increasing Treg cells in lung^26^, these two flavonoid inducing more Treg cells at a lower dose than glycyrrhizic acid. Licorice fractions contain these two flavonoids shown a significant potential to promote Treg cell induction and function, but the fractions without these two constituents didn’t show similar activities. These results suggested that isoliquiritigenin and naringenin could be the major constituents in licorice to promote Treg cell induction and enhance its function. Because isoliquiritigenin promoted Treg cells to almost maximum level *in vivo*, we couldn’t detect any additive Treg promoting effects with both isoliquiritigenin and naringenin treatment *in vivo.*

Though the licorice has been widely used as a drug to treat peptic ulcer, asthma, colitis and other inflammatory diseases for a long time, the molecular mechanism of licorice is not well understood. Recent study reported naringenin promote Treg cells as an AhR agonist^31^. Our study confirmed it and found licorice and its active fractions also could activate AhR signaling like naringenin, but isoliquiritigenin could not. These data indicated isoliquiritigenin might promote Treg cells by a different mechanism to naringenin. AKT-mTOR signaling has been found to suppress the induction of FOXP3 expression *in vitro* and *in vivo*. Manipulation of AKT-mTOR signaling activity by pharmacological compounds such as rapamycin and everolimus (Afinitor; Novartis) has been approved for clinical use in autoimmune and inflammatory diseases, such as inflammatory colitis^8^. In our study, the inhibition of AKT/mTOR signaling was observed in CD4^+^ T cells treated with isoliquiritigenin in Treg cell-inducing conditions, which was not detectable in CD4^+^ T cells treated with naringenin. Licorice extract and it active fraction was also found reducing activity of AKT, suggesting inhibition of AKT-mTOR signaling was another mechanism for licorice promoting Treg cells and suppressing inflammation.

The pivotal role of Treg cells in immune regulation has been widely appreciated. Transfer of Treg cells or increasing Treg cells induction have been proved to be an efficient therapeutic methods for autoimmune and inflammatory diseases in variety animal models. Oral administration of isoliquiritigenin, naringenin or Gly1 fraction ameliorated pathological symptoms of DSS-induced colitis. Our preliminary results suggest that isoliquiritigenin, naringenin and Gly1 fraction might serve as a potential therapeutic drug for inflammatory colitis or other autoimmune and inflammatory diseases, including inflammatory bowel disease, rheumatoid arthritis and multiple sclerosis.

## Methods

### Mice

C57BL/6 mice were obtained from the Shanghai Laboratory Animal Center (Chinese Academy of Sciences). Foxp3-IRES-GFP (Foxp3^*egfp*^) mice, which contain the enhanced green fluorescence protein (*egfp*) gene under the control of an internal ribosomal entry site (IRES) inserted downstream of the foxp3 coding region as described elsewhere, were provided by Dr honglin Wang (Shanghai Jiaotong University). All mice were maintained in pathogen-free conditions. Animal care and use were in accordance with the guidelines of the Institute of Biochemistry and Cell Biology, Chinese Academy of Sciences. All animal experimental procedures were approved and overseen by the Animal Care and Use Committee of the Shanghai Institute of Biochemistry and Cell Biology, Chinese Academy of Sciences.

### Reagents

FITC-conjugated anti-mouse CD4 (GK1.5; 11-0041), PE-Cy7-conjugated anti-mouse IFN-γ (XMG1.2; 25-7311), APC-conjugated anti-mouse Foxp3 (FJK-16s; 17-5773) were from eBioscience. APC-conjugated anti-mouse IL17A (TC11-18H10; 559502) were from BD Biosicence. Collagenase D (11088866001) was from Roche Diagnostics GmbH. Dispase (17105-041, GIBCO) was from Invitrogen Corporation. DNase I was from Worthington. Dextran Sulfate Sodium Salt (M.W. = 36,000–50,000, 160110) was purchased from MP Biomedicals, LLC.

### Primary CD4 T-cell purification, culture and *in vitro* differentiation

Naive CD4^+^ CD25^−^ T cells from the spleen of 8- to 12-week-old mice were purified by selection using magnetic cell sorting. For *in vitro* T helper (Th) cell differentiation assay, sorted cells were activated with anti-CD3e (2 ug/ml; 145-2C11, soluble; BD Pharmingen) and anti-CD28 (2 ug/ml; 37.51, soluble; BD Pharmingen) and were induced to differentiate into Th1 cells by supplementation with IL-12 (10 ng/ml; Peprotech) plus anti-IFN-γ (5 ug/ml, XMG1.2; BD Pharmingen); or into Th17 cells with transforming growth factor-β1 (TGF-b1; 3 ng/ml, Peprotech), IL-6 (30 ng/ml; eBioscience) and anti-IFN-γ. For inducing the differentiation of naive CD4^+^CD25^−^ T cells into Treg cells, cells were activated by plate-coating anti-CD3e (1 ug/ml) plus soluble anti-CD28 (1 ug/ml) and treated with TGF-β1 (1 ng/ml) and anti-IFN-γ. Cells stimulated in ‘neutral’ conditions (anti-IL-4, anti-IFN-γ but without additional cytokines) were considered to be ‘Th0’ cells.

### Flow Cytometry

For intracellular cytokine staining, cells were stimulated for 4 hr. with phorbol 12-myristate 13-acetate (50 ng/ml; P8139; Sigma) and ionomycin (1 μM; I3909; Sigma) in the presence of Brefeldin A (3 μg/ml; 00–4506; eBioscience). Then, cells were stained for surface molecules, fixed and made permeable with Fixation/Permeabilization Solution Kit (554714; BD Bioscience), and stained with fluorescence labeled antibodies. For analysis of intracellular Foxp3, cells were stained for surface markers, fixed and made permeable with Foxp3 Staining Buffer Set (00–5523; eBioscience), and stained with fluorescence labeled antibodies. Samples were run with FACSCalibur (BD Bioscience) and analyzed using FLOWJO software (Treestar Inc, Ashland, OR).

### *In vitro* suppression assay

Cells were isolated from the spleen of 8- to 12-week-old mice. Regulatory T cells were isolated by the CD4^+^CD25^+^Regulatory T Cell Isolation Kit (130-091-041, Miltenyi Biotec). CD4^+^CD25^−^ T cells were used as T conventional cells. T conventional cells were labelled with 1 μM CFSE (C34554, life technologies). Magnetically sorted Treg cells were cultured with T conventional cells (5 × 10^4^ cells) at different ratios in 96-well round bottom plates together with irradiated T-cell-depleted splenocytes (5 × 10^4^ cells) as antigen-presenting-cells and anti-CD3 (1 μg/ml). 96 hr. later, the suppression was assayed by FACS analysis for dilution of CFSE in gated conventional T cells.

### Colonic lamina propria lymphocyte isolation

To isolate lamina propria (LP) lymphocytes, colons were collected and opened longitudinally, and shaken with HBSS containing 5 mM EDTA at 37 °C with gentle shaking for 30 minutes to remove epithelial cells. Then the colons were cut into small pieces and incubated with PRMI 1640 containing 0.5 mg/ml collagenase D and 0.5 mg/ml Dispase and 40 mg/ml DNase I for 1 hr. at 37 °C with gentle shaking. The digested tissues were collected by filtering through a 40-μm cell strainer and washed with RPMI 1640 containing 2 mM EDTA. LP lymphocytes (LPLs) were resuspended in 5 ml of 40% Percoll and layered on top of 2.5 ml of 80% Percoll. LPLs were collected from the interface of the 40% and 80% gradient after centrifugation.

### Preparation and fractionation of extracts from licorice

The water extract of licorice from Inner Mongolia Autonomous Region in China (purchased from Jiangyin Tianjiang Pharmaceutical Co., Ltd) was dissolved in water and extracted by ethyl acetate for four times. The organic solvents were removed by vacuum evaporation and named Gly1. The water soluble fraction was chromatographed on silica gel with CHCl3-MeOH-H2O (9:1:0.1 to 0:1:0) and get the fraction Gly2, Gly3 and Gly4. The Gly1 fraction was chromatographed on silica gel with Petroleum ether-acetone (10:0 to 8:2), and get the fraction Gly17, Gly18, Gly19 and Gly20. The Gly18 fraction was chromatographed on silica gel for several times and get two chemicals, Gly27 and Gly28. Gly19 fraction was chromatographed and get two chemicals, Gly29 and Gly30. The Gly27 (liquiricidin), Gly28 (liquiritigenin), Gly29 (naringenin), Gly30 (isoliquiritigenin) was analyzed by NMR.

### DSS-induced colitis

C57BL/6 mice were given 2.5% (w/v) DSS in drinking water for 6 days. Licorice extracts, fractions or chemicals were dissolved in water and orally administrated every day, starting 7 days prior to the DSS treatment and continued to the end of the experiment. After induction of colitis, body weight and stool condition were analyzed on a daily basis. The disease severity was measured by percent weight loss, intestinal bleeding (no blood, occult blood, or gross blood), stool consistency (normal, loose stool or diarrhea) and colon length.

### Reverse transcription and quantitative real-time PCR

Total RNA was extracted with TRI Reagent (T9424; Sigma) according to the manufacturer’s instructions. Random hexamer primer and M-MLV Reverse Transcriptase (M5301; Promega) were used for reverse transcription. Real-time PCR was performed using JumpStart Taq ReadyMix (D7440; Sigma) supplemented with EvaGreen Dye (31000; Biotium) on a Stratagene Mx3000P (Agilent Technologies). Primers used to quantify HIF1α mRNA were 5′-ACCTTCATCGGAAACTCCAAAG-3′(forward) and 5′-CTGTTAGGCTGGGAAAAGTTAGG-3′(reverse); SLC2A1 mRNA were 5′-CAGTTCGGCTATAACACTGGTG-3′(forward) and 5′-GCCCCCGACAGAGAAGATG-3′(reverse); CYP1A1 mRNA were 5′-GACCCTTACAAGTATTTGGTCGT-3′(forward) and 5′-GGTATCCAGAGCCAGTAACCT-3′(reverse).

### Western blot

CD4^+^CD25^−^ T cells were stimulated under conditions as described. Cells were collected and lysed for SDS-PAGE. Proteins were blotted onto nitrocellulose membrane (GE Healthcare). Blots were probed with the following antibodies: phosphor-Akt (Ser473) (4060, Cell Signaling), Akt (pan) (4691, Cell signaling), phosphor-Erk1/2 (9101, Cell Signaling), Erk1/2 (sc-94, Santa Cruz), phosphor-P70 S6 kinase (Thr389) (9234, Cell Signaling), P70 S6 kinase (2708, Cell Signaling), phosphor-S6 Ribosomal (Ser240/242) (4858, Cell, Signaling). The IRDye800CW-conjugated secondary antibody (Rockland) was then added. Data were assessed by the Odyssey Infrared Imaging System (Li-COR Bioscience, Lincoln, NE).

### Thin layer chromatograph and NMR

1D NMR were recorded on AVANCE III-600 spectrometers (Bruker, Bremerhaven, Germany). Silica gel (200–300 mesh) for column chromatography was purchased from Qingdao Makall Chemical Company (Makall, Qingdao, China). Sephadex LH-20 (20–50 μm) for chromatography was obtained from Pharmacia Fine Chemical Co., Ltd. (Pharmacia, Uppsala, Sweden). Fractions were monitored by thin layer chromatography (TLC) (GF254, Qingdao Haiyang, Qingdao, China), and spots were visualized by heating silica gel plates sprayed with 10% H2SO4 in EtOH.

The compounds Gly29 and Gly30 were identified as naringenin^29^ and isoliquiritigenin^30^ by comparing their NMR data with literatures.

In addition, the structures were also confirmed by the thin layer chromatography (TLC) method. The TLC was performed on plates coated with silica gel F254 and developed with ethyl acetate-chloroform (2:8, v/v) and acetone-petroleum ether (3:7, v/v), respectively. Both compounds Gly29, Gly30 and corresponding standard compounds (naringenin and isoliquiritigenin) had the identical Rf values on TLC from two mobile phases mentioned above.

Therefore, the structure of compounds Gly29 and Gly30 were established as naringenin and isoliquiritigenin, respectively.

### Statistical analysis

Data are presented as means ± SEM. Differences between data sets were analyzed by Mann-Whitney U tests, or one-way ANOVA followed by Bonferroni’s test. The two-way ANOVA and Mann-Whitney U test were used to assess the differences in the DSS-induced colitis mice between groups. *P*-value < 0.05 was considered statistically significant. Graphs were created in GraphPad Prism software.

## Additional Information

**How to cite this article**: Guo, A. *et al.* Promotion of regulatory T cell induction by immunomodulatory herbal medicine licorice and its two constituents. *Sci. Rep.* **5**, 14046; doi: 10.1038/srep14046 (2015).

## Supplementary Material

Supplementary Information
