# Supplementary material for: Promotion of regulatory T cell induction by immunomodulatory herbal medicine licorice and its two constituents
Source: Sci Rep. 2015 Sep 15;5:14046. doi: 10.1038/srep14046 (PMC4570190; doi:10.1038/srep14046)

Promotion of regulatory T cell induction by immunomodulatory herbal medicine licorice and its two constituents

Ao Guo[1, 2], Dongming He[2, 4], Hong-Bo Xu[3], Chang-An, Geng[3], Jian Zhao[2*]

[1]School of Life Sciences, University of Science and Technology of China, Hefei, Anhui 230026, China

[2]State Key Laboratory of Cell Biology, Institute of Biochemistry and Cell Biology, Shanghai Institute for Biological Sciences, Chinese Academy of Sciences, Shanghai 200031, China

[3]State Key Laboratory of Phytochemistry and Plant Resources in West China, Kunming Institute of Botany, Chinese Academy of Sciences, Kunming 650201, China

[4] School of Life Science and Technology, ShanghaiTech University, Shanghai 201210, China

SUPPLEMENTARY FIGURE LEGENDS

Supplementary Figure 1

Effects of nine traditional Chinese medicines on Treg cell induction *in vitro*.

Naive $CD4^+$ T cells were stimulated with soluble anti-CD3, anti-CD28 monoclonal antibodies and TGFβ (1 ng/ml) under the indicated concentrations of nine traditional Chinese medicines extract and analyzed by FACS.

Supplementary Figure 2

Licorice extract and its fractions didn't affect Th1 and Th17 cell differentiation *in vitro.*

(a) Naive $CD4^+$ T cells were stimulated with soluble anti-CD3, anti-CD28 monoclonal antibodies and IL-12 (10ng/ml) under the indicated concentrations of licorice extract and analyzed by FACS. (b) Naive $CD4^+$ T cells were stimulated with soluble anti-CD3, anti-CD28, anti-IFN-γ monoclonal antibodies, TGFβ (3ng/ml) and IL-6 (30ng/ml) under the indicated concentrations of licorice extract and analyzed by FACS. Results are expressed as means ± SEM and are representative of three experiments. *$P< 0.05$, **$P< 0.01$ and ***$P< 0.001$. One-way ANOVA was used to analyze these data.

Supplementary Figure 3

Licorice extract and its fractions didn't affect Th1 and Th17 cells induction *in vivo*.

(a) C57BL/6 mice were orally administrated with licorice or Gly1 fractions for two weeks, Treg cells were analyzed by FACS. (b) C57BL/6 mice were orally

administrated with licorice for two weeks, Th1 and Th17 cells were analyzed by FACS. (c) C57BL/6 mice was orally administrated with Gly1 fraction for two weeks, Th1 and Th17 cells were analyzed by FACS. Results are expressed as means ± SEM and are representative of three experiments. *P< 0.05, **P< 0.01 and ***P< 0.001. Man-Whitney U test was used to analyze these data.

Supplementary Figure 4

Th1 and Th17 cytokines profile with licorice and its two active constituents *in vitro*

(a) Quantitative PCR analyses of Th1 cytokines expression in $CD4^+$ T cells under Th1-inducing conditions with licorice, Gly1 fraction, isoliquiritigenin or naringenin. (b) Quantitative PCR analyses of Th17 cytokines expression in $CD4^+$ T cells under Th17-inducing conditions with licorice, Gly1 fraction, isoliquiritigenin or naringenin. Results are expressed as means ± SEM and are representative of three experiments. *P< 0.05, **P< 0.01 and ***P< 0.001, as determined by one-way ANOVA followed by Bonferroni's test.

Supplementary Figure 5

Gly1 sub-fraction promote Treg cells induction and function *in vitro*.

(a) Naive $CD4^+$ T cells were stimulated with immobilized anti-CD3, soluble anti-CD28 monoclonal antibodies and TGFβ (1 μg/ml) under the indicated concentrations of licorice extract and analyzed by FACS. (B) $CD4^+CD25^+$ Treg cells untreated or treated with Gly1 sub-fractions were incubated with CFSE labelled $CD4^+CD25^-$ Tconv cells in an in vitro suppression assay. The suppression was assayed by FACS analysis for dilution of CFSE in gated responder T cells. Results are expressed as

means ± SEM and are representative of three experiments. *P< 0.05, **P< 0.01 and ***P< 0.001. One-way ANOVA or two-way ANOVA was used.

Supplementary Figure 6

Thin layer chromatography analysis of chemicals isolated from Gly18 and Gly19

(a-c) TLC analysis of Gly29 and Gly30, two chemicals isolated from Gly18 and Gly19 fraction with naringenin and isoliquiritigenin standard. (d, e) TLC analysis of Gly1, Gly17, Gly18, Gly19, Gly20 with naringenin and isoliquiritigenin standard.

Supplementary Figure 7

NMR Spectroscopy of isoliquiritigenin and naringenin isolated from licorice fractions

The structures of compounds Gly29 and Gly30 were identified as naringenin and isoliquiritigenin by comparing their NMR data with literatures

Supplementary Figure 8

Cytotoxicity of licorice and its active constituents.

Naive $CD4^+$ T cells were cultured with licorice extracts, Gly1 fraction, isoliquiritigenin or naringenin for 24 hours and cell viability was tested.

Supplementary Figure 9

Naringenin and isoliquiritigenin didn't regulate Th1 and Th17 cell generation *in vivo*

C57BL/6 mice were orally administrated with isoliquiritigenin or naringenin for two weeks, Treg cells in spleen and lymph node were analyzed by FACS.

Supplementary Figure 10

Full-length blots for Figure 5.

(a) Full-length blots for Figure 5a. (b) Full-length blots for Figure 5c. (c) Full-length blots for Figure 5d.

Supplementary Figure 1

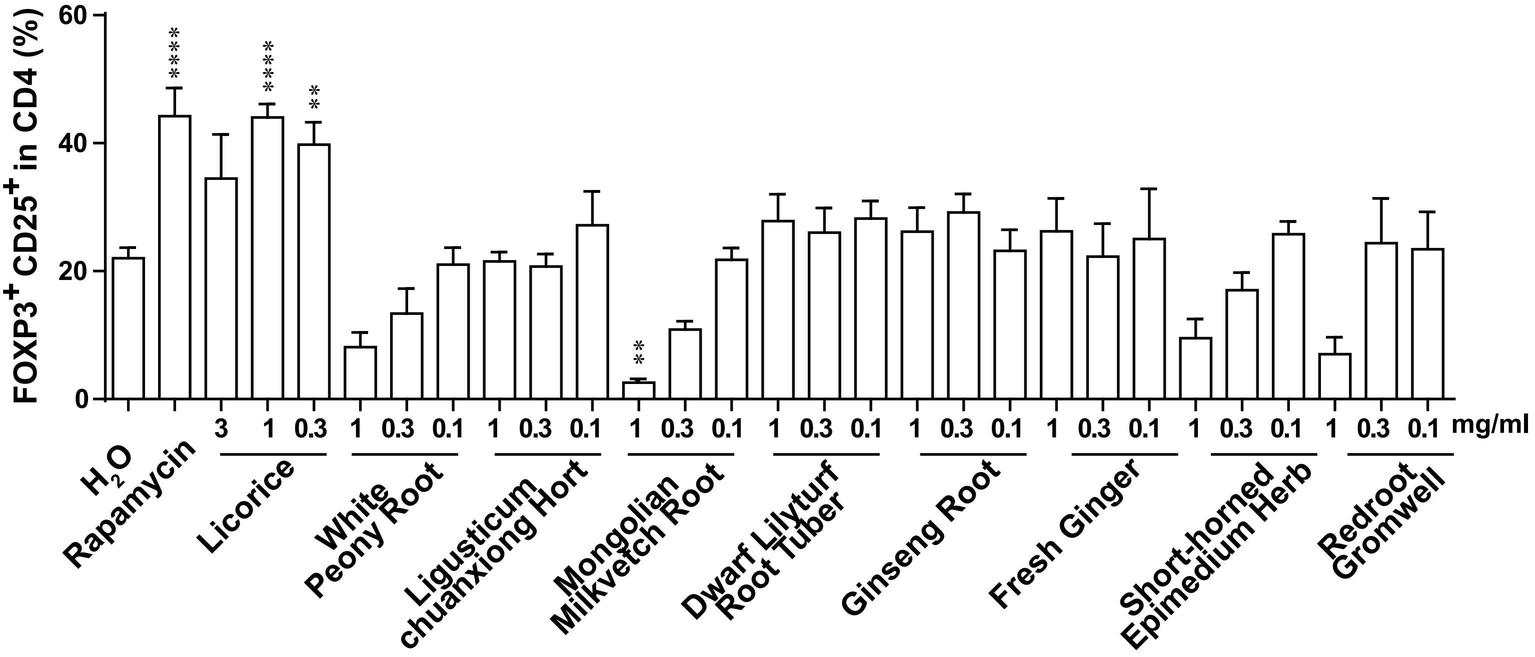

Supplementary Figure 2

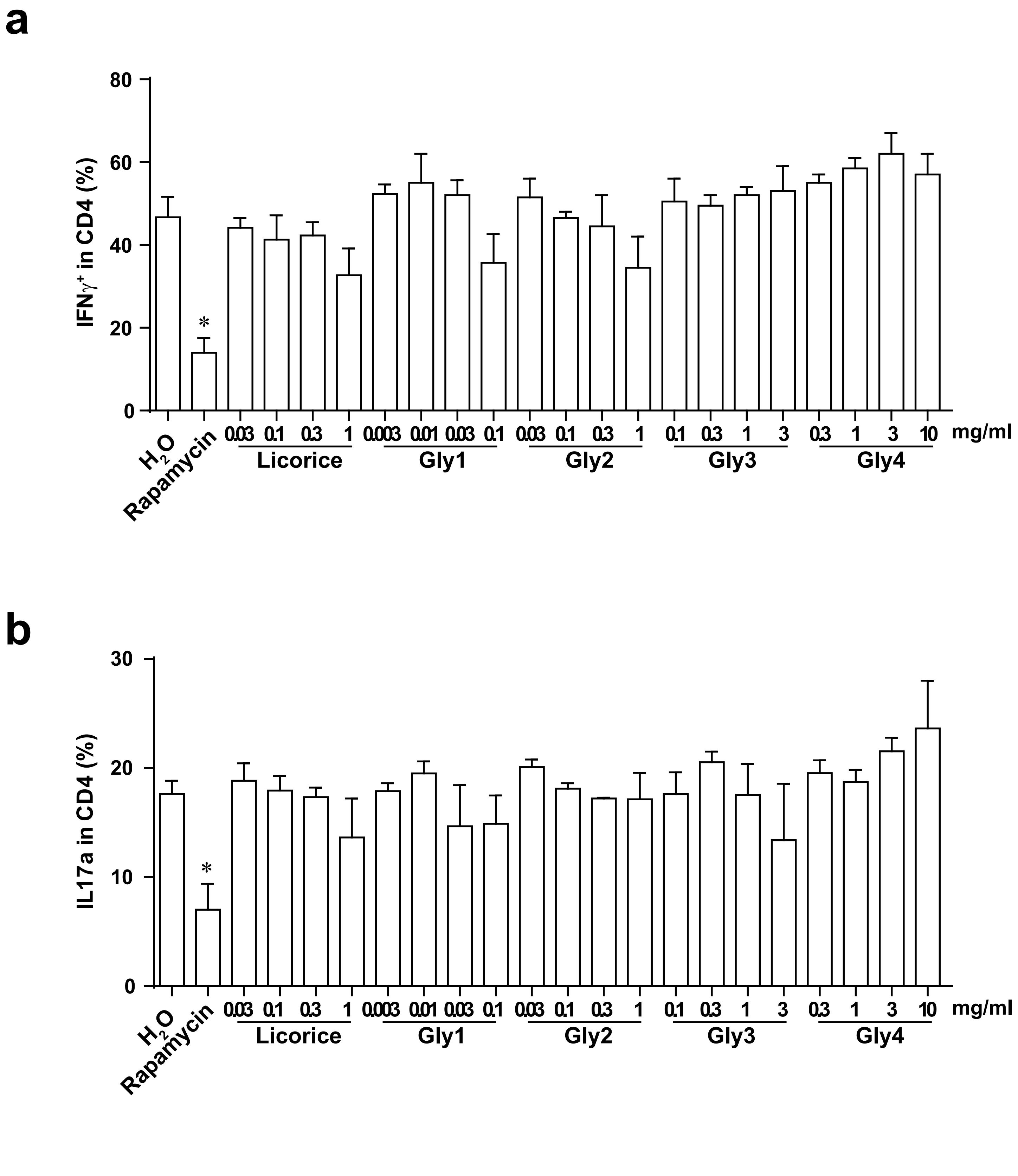

Supplementary Figure 3

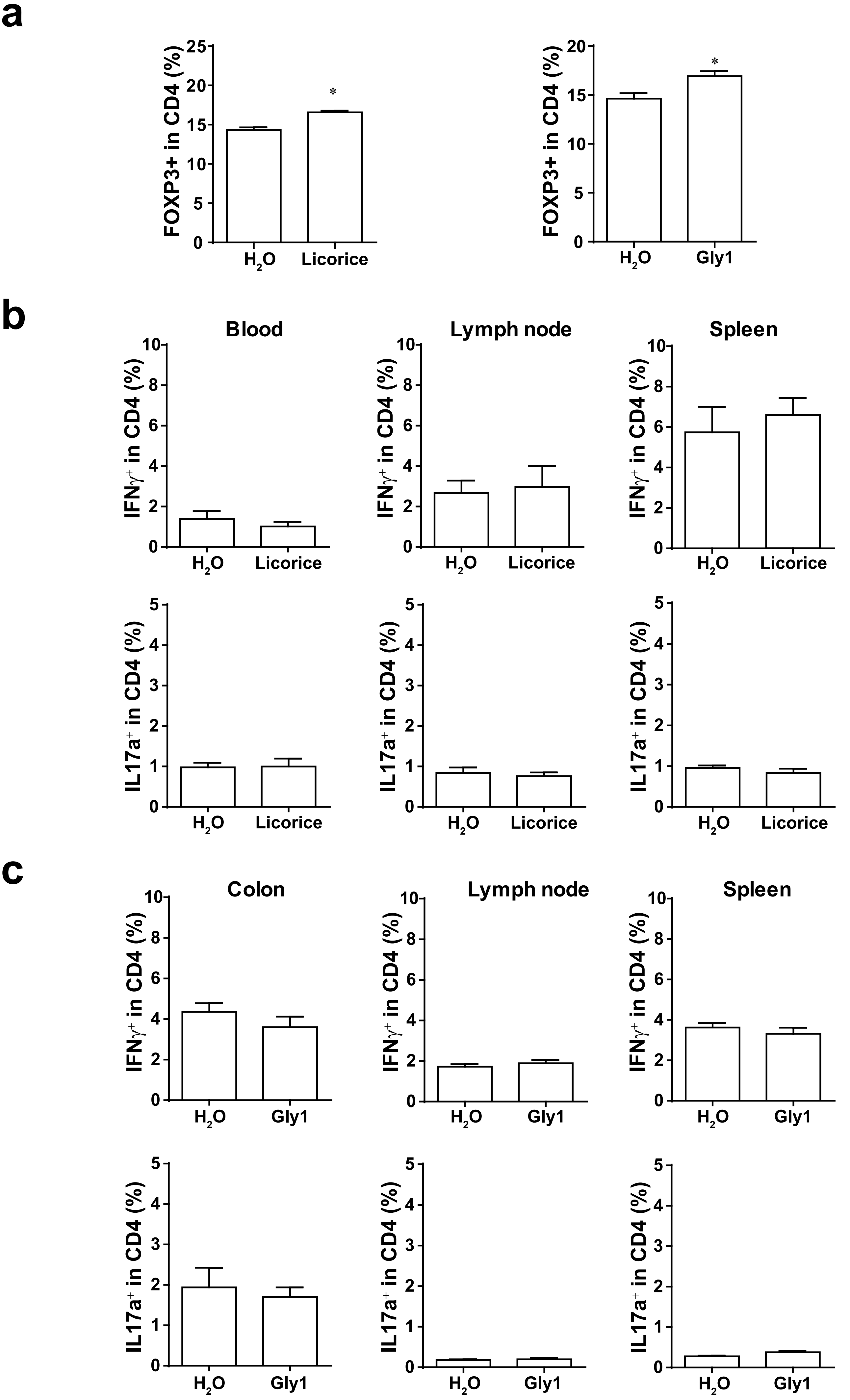

Supplementary Figure 4

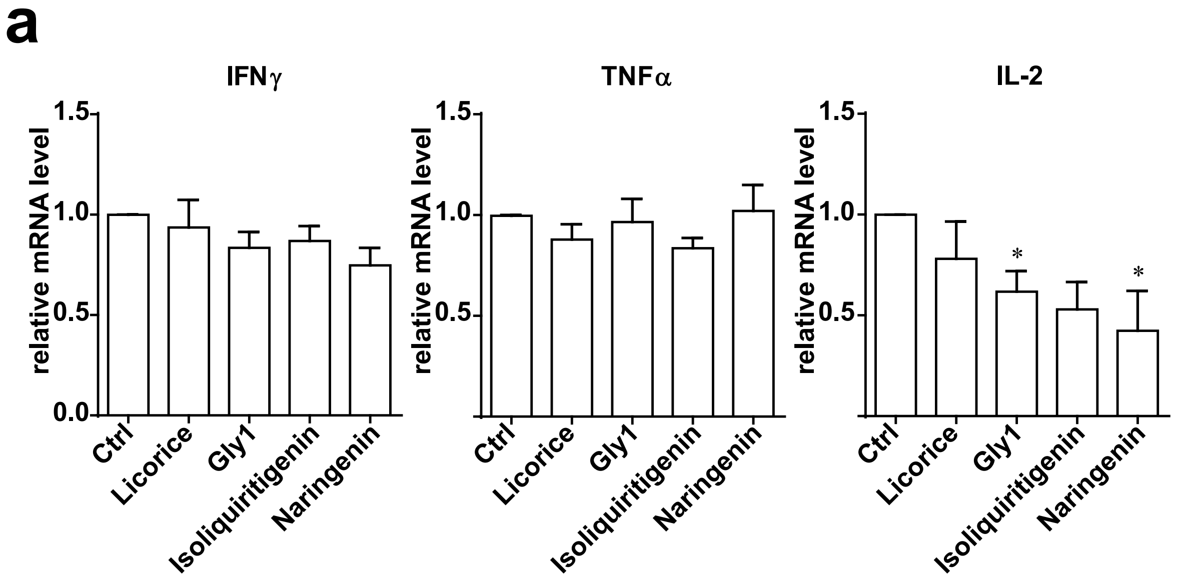

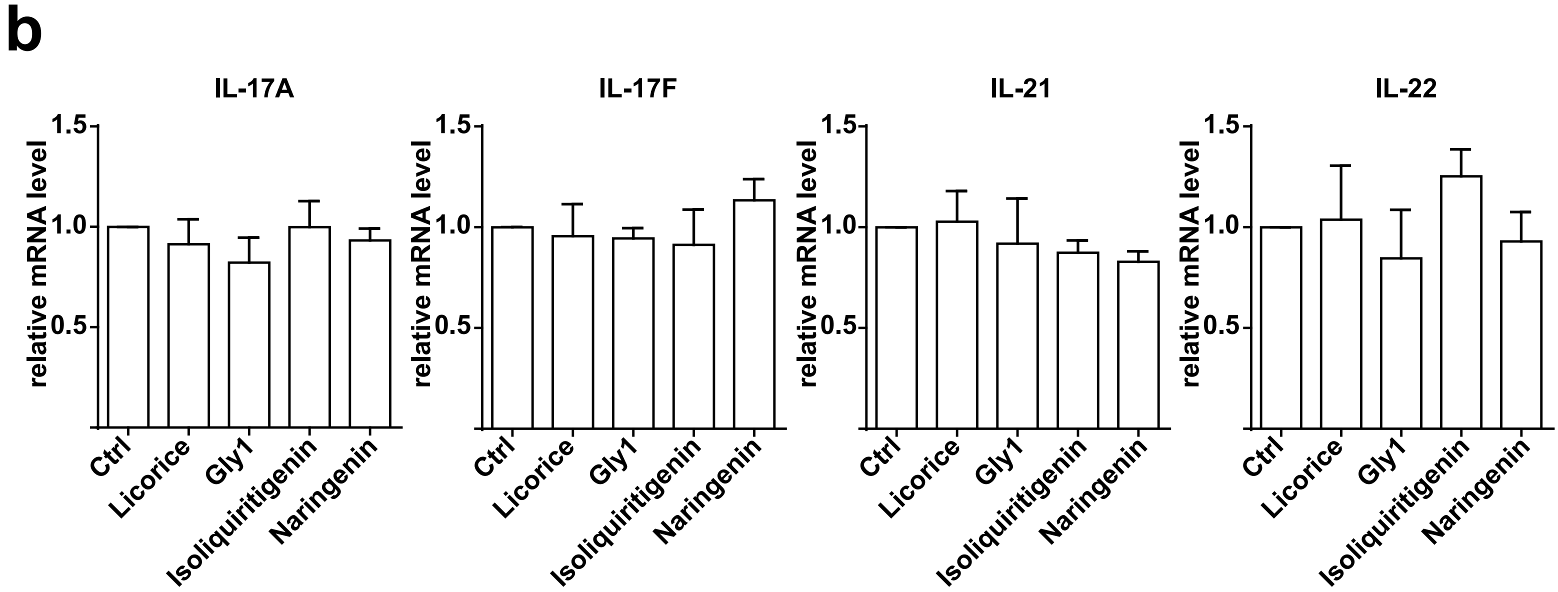

Supplementary Figure 5

a

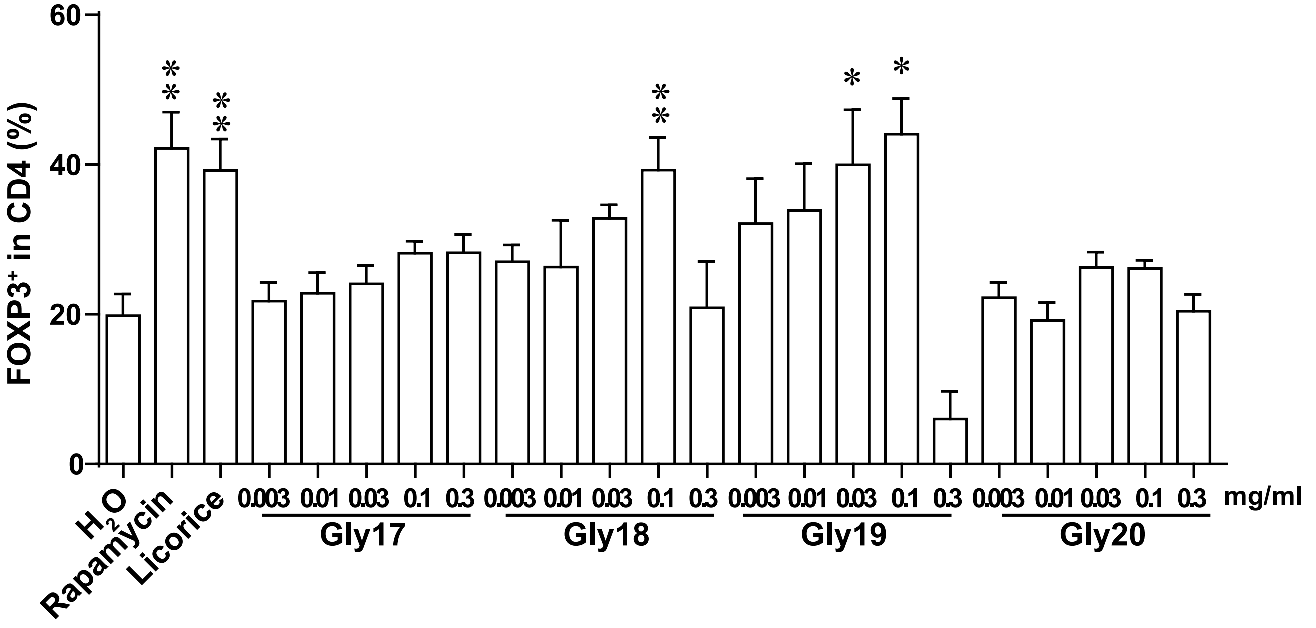

b

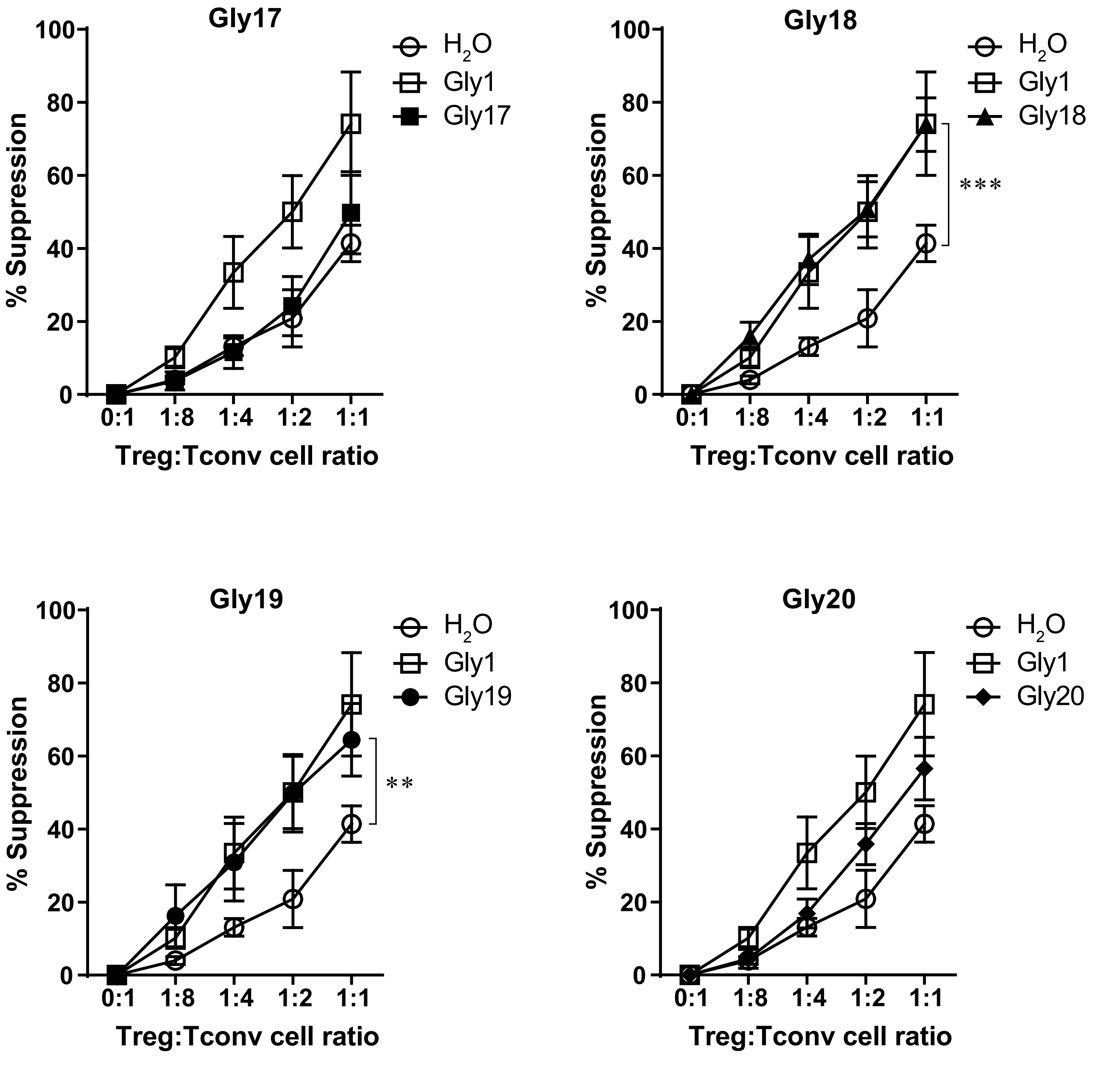

Supplementary Figure 6

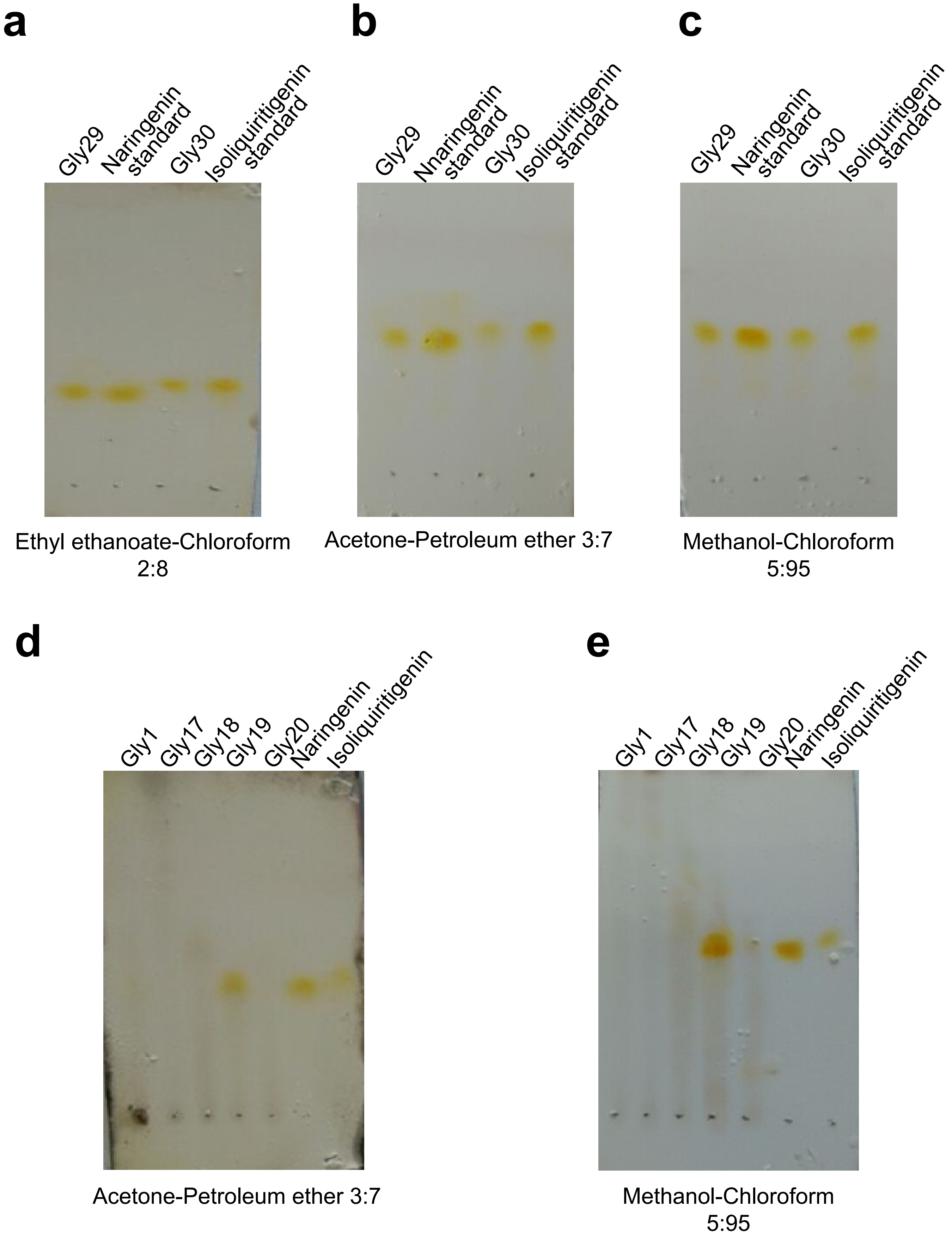

Supplementary Figure 7

Position	Gly 29[1]		Gly 30[2]	
1			127.4	
2	79.9	dd, 5.44, 13.2, 3.2	131.7	7.75, d, 8.4
3	43.4	H-3a: 2.72, dd, 16.8, 3.2<br>H-3b: 3.04, dd, 16.8, 13.2	116.7	6.47, d, 8.4
4	197.2		160.9	
5	165.2		116.7	6.47, d, 8.4
6	96.7	5.94, s	131.7	7.75, d, 8.4
7	167.3			
8	95.8	5.94, s		
1’	130.7		114.4	
2’	129.0	7.38, d, 8.3	165.6	
3’	116.1	6.88, d, 8.3	103.6	6.37, s
4’	158.7		167.5	
5’	116.1	6.88, d, 8.3	108.6	6.47, d, 9.0
6’	129.0	7.38, d,8.3	133.2	8.11, d, 9.0
α	103.1		118.2	7.76, d, 14.4
β	164.3		145.1	7.82, d, 14.4
C=O			192.7	

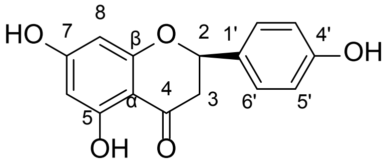

Gly29(Naringenin)

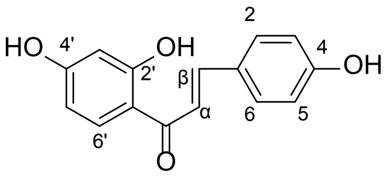

Gly30(Isoliquiritigenin)

[1] Khalid S A, Yagi S M, Khristova P, et al. (+)-Catechin-5-galloyl Ester as a novel natural polyphenol from the bark of *Acacia nilotica* of Sudanese Origin. Planta medica, 1989, 55(06): 556-558.

[2] Aida K, Tawata M, Shindo H, et al. Isoliquiritigenin: a new aldose reductase inhibitor from *Glycyrrhizae radix*. Planta medica, 1990, 56(03): 254-258.

Supplementary Figure 8

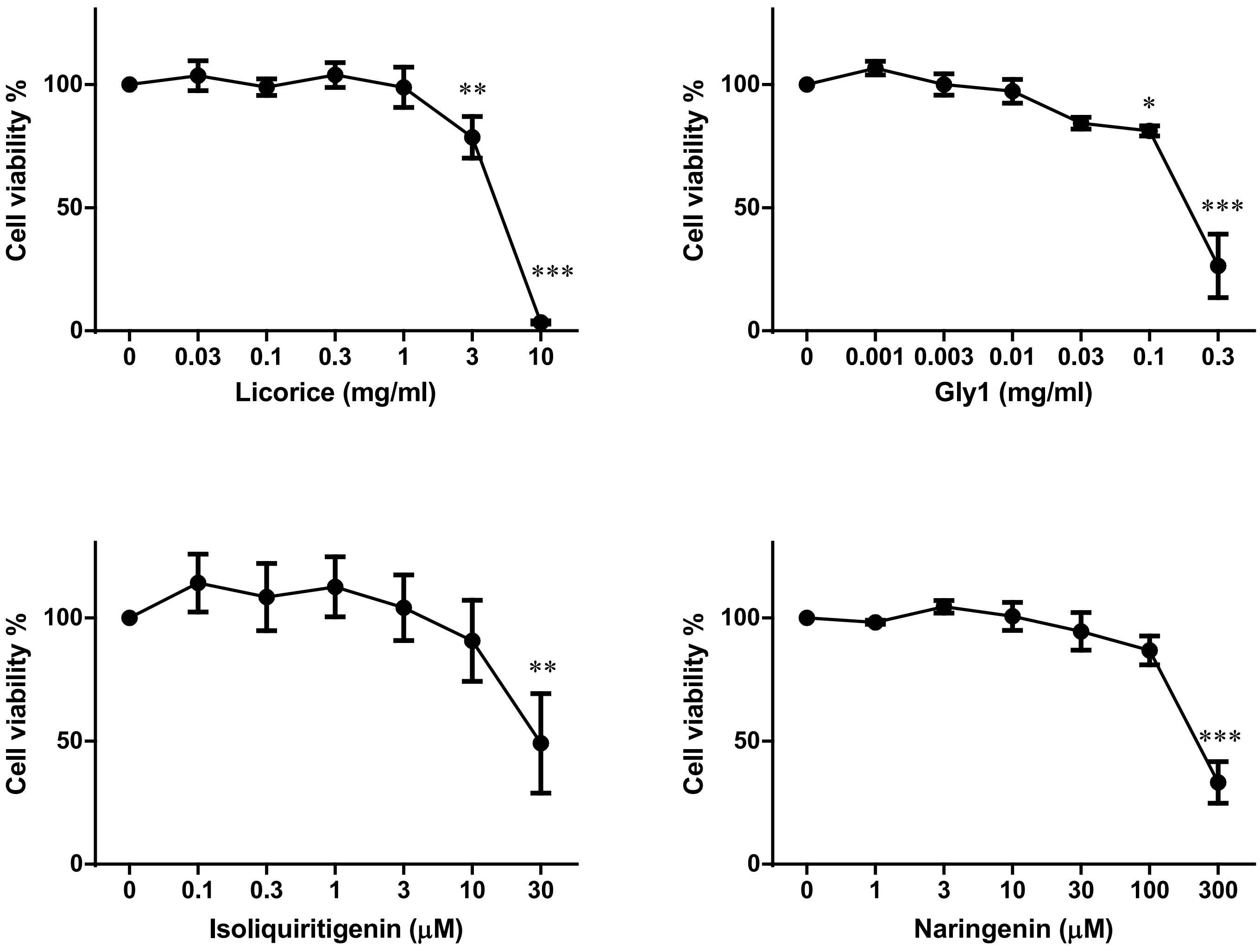

Supplementary Figure 9

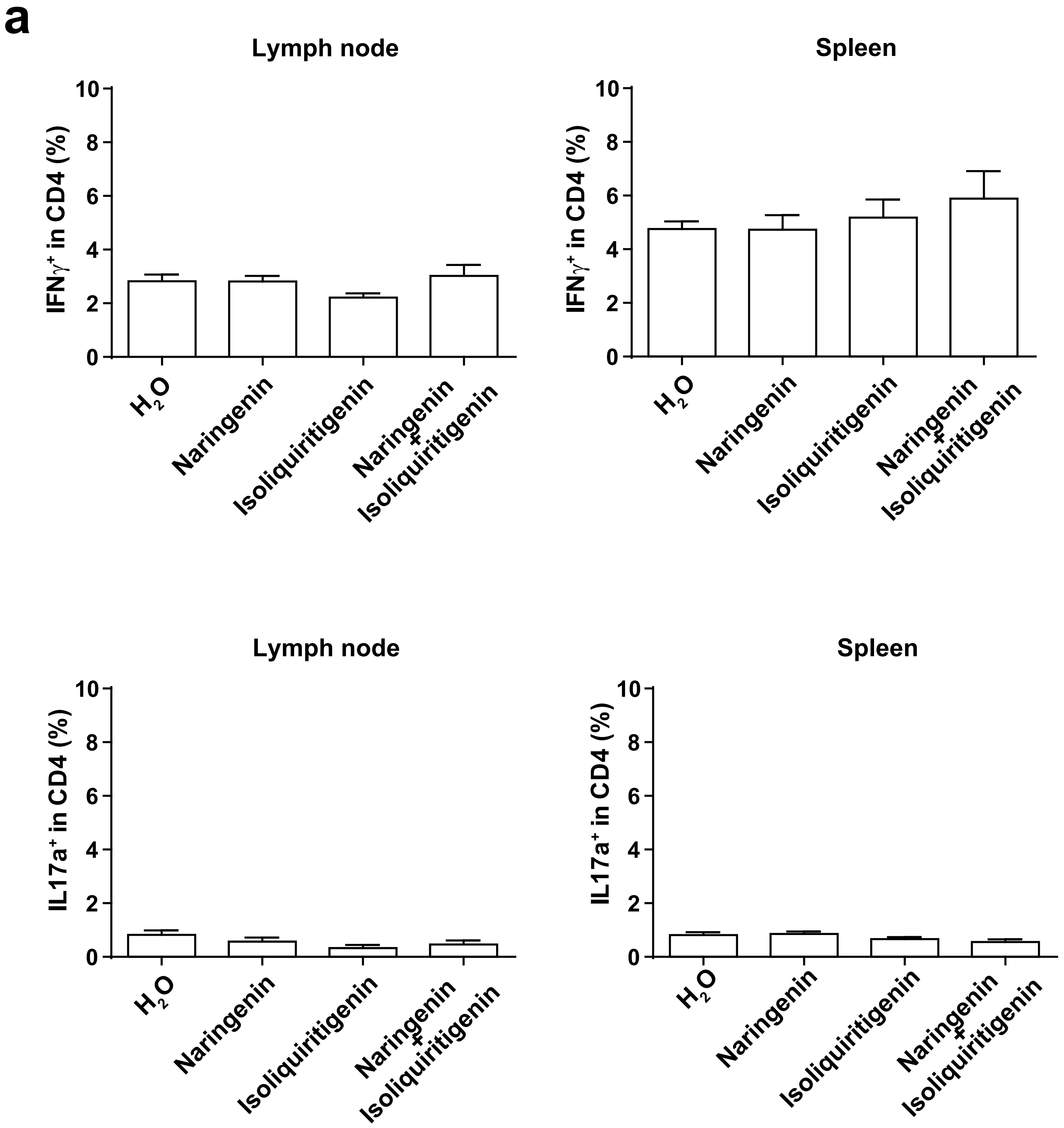

Supplementary Figure 10

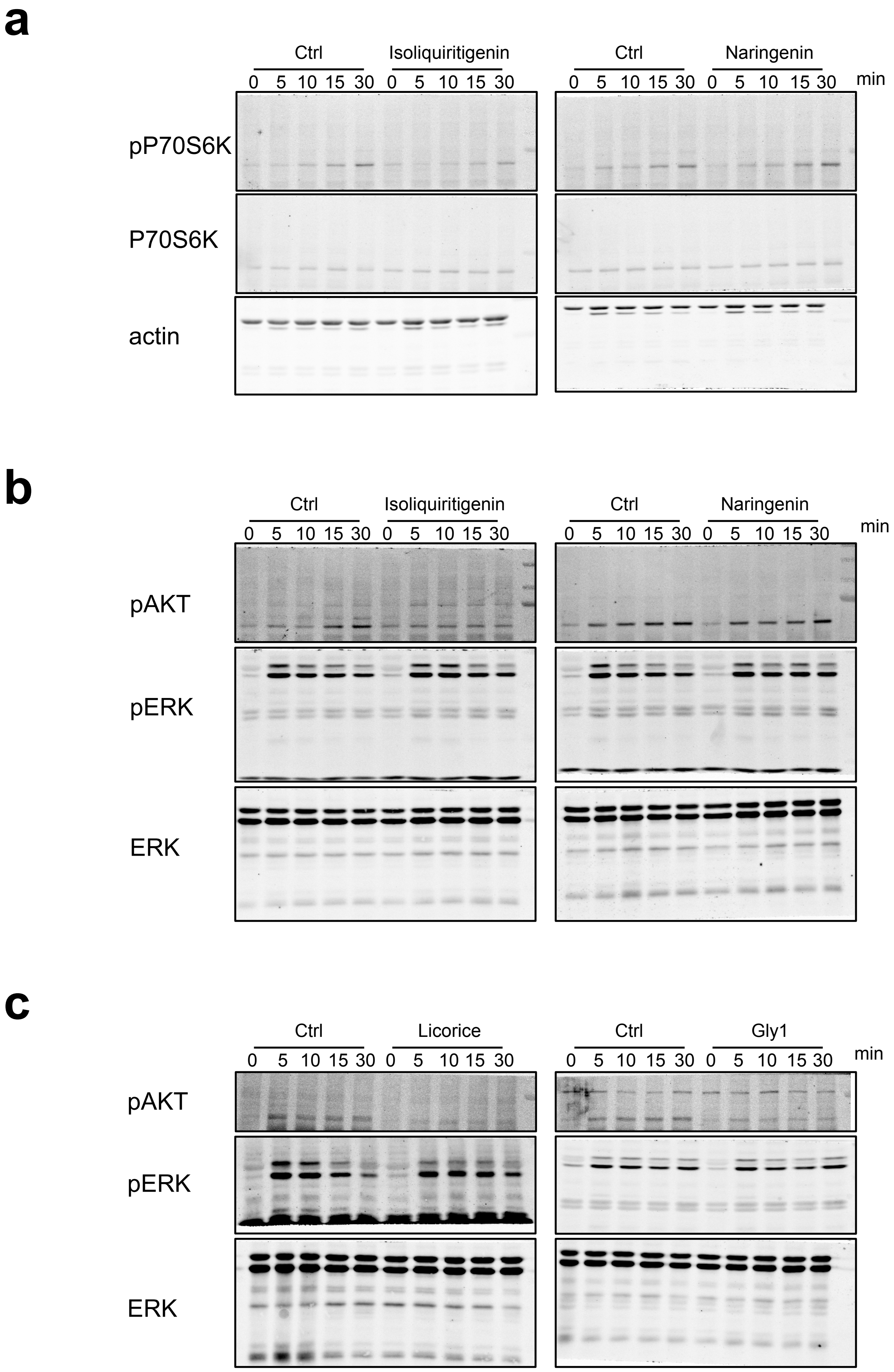

Supplement: Supplementary Information [file srep14046-s1.pdf]
